# Comparison of 1-year therapeutic effect of ranibizumab and bevacizumab for myopic choroidal neovascularization: a retrospective, multicenter, comparative study

**DOI:** 10.1186/1471-2415-14-69

**Published:** 2014-05-21

**Authors:** Dong Min Cha, Tae Wan Kim, Jang Won Heo, Se Joon Woo, Kyu Hyung Park, Hyeong Gon Yu, Hum Chung

**Affiliations:** 1 Department of Ophthalmology, Jeju National University College of Medicine, Jeju, Korea; 2 Department of Ophthalmology, Seoul National University College of Medicine, Seoul, Korea; 3 Department of Ophthalmology, Seoul Metropolitan Government Seoul National University Boramae Medical Center, #395 Shindaebang-2-dong, Dongjak-gu, Seoul 156-707, Korea; 4 Department of Ophthalmology, Seoul National University Bundang Hospital, Seongnam, Korea

**Keywords:** Anti-VEGF (vascular endothelial growth factor), Bevacizumab, Myopic choroidal neovascularization, Ranibizumab

## Abstract

**Background:**

To compare the long-term efficacy of ranibizumab versus bevacizumab for myopic choroidal neovascularization (CNV).

**Methods:**

This was a retrospective, multicenter, comparative, non-randomized study of 64 consecutive patients with myopic CNV treated with ranibizumab (22 patients) or bevacizumab (42 patients). Best-corrected visual acuity (BCVA) and central foveal thickness (CFT) on optical coherence tomography were evaluated before and after treatment. All the patients were followed for at least 12 months.

**Results:**

BCVA (logarithm of the minimal angle of resolution) improved from 0.63 ± 0.30 to 0.43 ± 0.27, 0.41 ± 0.37, 0.40 ± 0.39, 0.39 ± 0.43, and 0.39 ± 0.42 at 1, 2, 3, 6, and 12 months after treatment in the ranibizumab group, and from 0.67 ± 0.28 to 0.52 ± 0.31, 0.49 ± 0.31, 0.47 ± 0.31, 0.42 ± 0.32, and 0.46 ± 0.43 in the bevacizumab group (all *P* < 0.05 compared with baseline BCVA in each group). CFT decreased by 20.21%, 19.58%, and 22.43% from the baseline 304 ± 76 μm at 3, 6, and 12 months after treatment in the former group, and by 15.20%, 15.67%, and 15.56% from the baseline 297 ± 62 μm in the latter group (all *P* < 0.05 compared with baseline CFT in each group). BCVA improvement and CFT reduction did not statistically differ when compared at the same periods from treatment between 2 groups. Neither ocular nor systemic safety problems appeared during follow up.

**Conclusions:**

This study showed a similar functional and anatomical improvement after treatment of ranibizumab and bevacizumab for myopic CNV over a 12-month follow-up period.

## Background

Myopic choroidal neovascularization (CNV) is the most common macular complication of pathologic myopia, with reported incidence ranging from 4–11% in highly myopic eyes [1,2]. Myopic CNV has typical characteristics including small size, more often subfoveal or juxtafoveal location, absence or minimal presence of subretinal fluid, and hemorrhage in the background of tigroid fundus appearance. Early consideration of myopic CNV is important because it often affects young people and its natural course without treatment is poor [1,2]. Various treatment protocols including direct photocoagulation, macular translocation, and photodynamic therapy (PDT) have been proposed; however these treatments for myopic CNV have not been satisfactory in the long term. In particular, PDT did not show a statistically significant treatment benefit for myopic CNV compared with a placebo group in the 2-year outcome in spite of its positive effect at 12 months in the Verteporfin in Photodynamic (VIP) trial [3,4].

The advent of anti-vascular endothelial growth factor (anti-VEGF) agents has changed the paradigm of various retinal diseases. Several reports have demonstrated the effectiveness of anti-VEGF agents including ranibizumab and bevacizumab for myopic CNV [5-13]. However, only a few studies have directly compared the effectiveness of treatment for myopic CNV between ranibizumab and bevacizumab [14-17]. Gharbiya et al. [14] reported that ranibizumab and bevacizumab had similar functional and anatomical effects on myopic CNV at 6-month follow up. Iacono et al. reported that these two drugs showed similar visual improvement over an 18-month follow-up in subfoveal CNV, although ranibizumab achieved greater efficacy than bevacizumab in terms of the mean number of injections administered [16]. Here, we compared functional and anatomical treatment effectiveness for myopic CNV between these 2 anti-VEGF agents over a 12-month follow-up period after treatment.

## Methods

One hundred twenty two eyes of 110 patients who were diagnosed with myopic CNV from 2007 to 2009 at 3 tertiary medical centers (Seoul National University Hospital, SNUH; Seoul National University Bundang Hospital, SNUBH; and Seoul National University Boramae Medical Center, SNUBMC) were retrospectively chart-reviewed in 2010, and 66 eyes of 64 patients satisfying inclusion and exclusion criteria were included in the study. The study protocol was approved by the Institutional Review Boards of the 3 hospitals and adhered to the tenets of the Declaration of Helsinki.

Inclusion criteria were as follows: (1) axial length more than 26 mm or spherical equivalent less than -6.0 Dsph (if patients had a history of cataract operation or refractive surgery, preoperative spherical equivalent was applied); (2) pathologic myopia classified as M2 or more according to the criteria of Avila et al. [18]; (3) myopic CNV that was newly diagnosed without prior treatment history; (4) pretreatment best-corrected visual acuity (BCVA) between 20/500 and 20/30; and (5) 12 or more months of follow up from treatment. Exclusion criteria were as follows: (1) history of treatment using photodynamic therapy or any intravitreal injection for myopic CNV before enrollment; (2) history of intraocular surgery except for cataract operation; (3) cataract operation which was performed less than 6 months before enrollment; (4) any other ocular disorder decreasing visual acuity such as corneal opacity, advanced cataract, and optic neuropathy; (5) cataract operation or Nd-YAG capsulotomy for after-cataract during the follow-up period; (6) BCVA less than 20/500 or more than 20/30; (7) follow-up period less than 12 months.

A thorough ophthalmic examination was performed in all patients. BCVA was measured using a Snellen chart and auto-refraction. Diagnosis of myopic CNV was confirmed when fluorescein angiography (FA; Topcon, Tokyo, Japan) revealed increased leakage over foveal area in the late phase compared with the early phase and the existence of neovascular lesion or subretinal fluid was observed with optical coherence tomography (OCT). The location of myopic CNV, which was classified into subfoveal, juxtafoveal, or extrafoveal one, was determined using both FA and fundus photography (Topcon, Tokyo, Japan). Three OCT modalities were used to evaluate central foveal thickness (CFT): Stratus OCT (Carl Zeiss Meditec, Dublin, CA), Cirrus OCT (Carl Zeiss Meditec, Dublin, CA), and Spectralis OCT (Heidelberg Engineering, Heidelberg, Germany). The same OCT modality was always used in each patient to measure CFT so that the error arising from changing OCT modalities could be minimized.

After explaining the patients the pros and cons of two anti-VEGF agents sufficiently, one anti-VEGF agent between bevacizumab (Avastin; Genetech, San Francisco, California, USA) and ranibizumab (Lucentis; Genentech, San Francisco, California, USA) was selected by the patients. A volume of 0.05 mL (1.25 mg bevacizumab or 0.5 mg ranibizumab) was injected into the vitreous cavity using a 30-gauge needle after topical anesthesia was induced by 1 drop of 0.5% proparacaine hydrochloride (Alcaine, Alcon Laboratories, Fort Worth, TX, USA) and povidone-iodine was applied inside the cul-de-sac.

All the patients followed 1 + PRN (Pro Re Nata) protocol. They were followed at 4-week intervals after the first injection. At visits, BCVA, thorough ophthalmic examination, FA, and OCT were performed. When intraretinal or subretinal fluid remained on OCT or fluorescein leakage was persistent on FA, an additional intravitreal injection of anti-VEGF agent was performed to the first injection. When the lesion subsided totally, the follow-up period was lengthened from 4 weeks to 2–3 months without treatment. All the patients were followed for at least 12 months from the first visit.

The primary outcome measures were changes in BCVA from baseline to 1, 2, 3, 6, and 12 months after treatment and the decreased rate (%) of the CFT from baseline to 3, 6, and 12 months after treatment in the bevacizumab and ranibizumab groups. Snellen visual acuity was converted to the logarithm of the minimum angle of resolution (logMAR). The secondary outcome measures were as follows: (1) the proportions of patients who gained or lost 2 or more lines of Snellen visual acuity at 6 and 12 months after treatment compared with baseline BCVA, (2) the proportions of patients in whom CFT was increased or decreased by 10% or more at 6 and 12 months in comparison with baseline CFT, (3) the rates at which FA revealed leakage at the late phase at 3, 6, and 12 months after treatment, (4) recurrence rates during 12 months after treatment, and (5) any complications associated with intravitreal injection over the follow-up period.

We used independent t-test, paired t-test, Pearson’s chi-square test, Mann–Whitney test, and Wilcoxon signed rank test to analyze demographic data and outcome measures. Statistical analyses were performed using SPSS for Windows (Ver. 18.0, Statistical Package for the Social Sciences, SPSS Inc., Chicago, IL). *P* values < 0.05 were considered significant.

## Results

Demographic data for the ranibizumab and bevacizumab groups are summarized in Table 1. The 64 patients comprised 42 patients in SNUH, 18 patients in SNUBH, and 4 patients in SNUBMC. No statistical differences between groups were observed for age, sex, laterality, refraction, axial length, follow-up period, lens status, pretreatment logMAR BCVA, location of CNV, or CFT.

**Table 1:** Patients demographics between ranibizumab and bevacizumab groups

**Parameters**	**Ranibizumab**	**Bevacizumab**	***P *** value*
Number	22 patients 23 eyes	42 patients 43 eyes	
Age (years)	50.48 ± 12.38	55.70 ± 12.78	0.12
Gender (male:female)	3:19	13:29	0.09
Laterality (right:left)	16:7	24:19	0.28
Refraction (diopter)	-12.34 ± 5.05	-14.85 ± 6.21	0.32
Axial length (mm)	30.28 ± 1.32	30.45 ± 1.51	0.87
Follow-up period (month)	22.87 ± 9.10	22.44 ± 9.41	0.86
Lens status			0.76
Phakia	16	25	
Pseudophakia or aphakia	7	18	
BCVA (logMAR)	0.63 ± 0.30	0.67 ± 0.28	0.59
Location of CNV			0.71
Subfoveal	15 (65%)	30 (70%)	
Juxtafoveal	8 (35%)	13 (30%)	
CFT (μm)	304 ± 73	287 ± 71	0.40

*CNV* choroidal neovascularization; *BCVA* best-corrected visual acuity; *logMAR* logarithm of the minimal angle of resolution; *CFT* central foveal thickness.

*Independent t-test or Pearson’s chi-square test were used.

**Figure 1:**
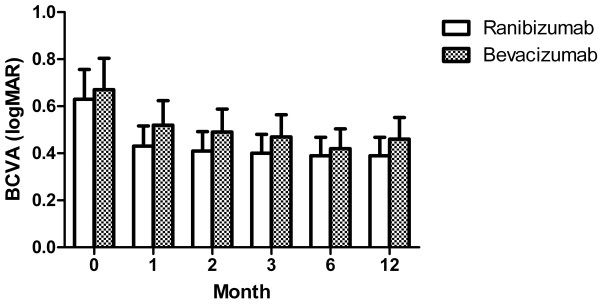
**Change of BCVA after treatment of ranibizumab and bevacizumab for myopic choroidal neovascularization.** Graph shows serial changes in best-corrected visual acuity (BCVA, logarithm of the minimal angle of resolution (logMAR)) from baseline to 12 months after treatment. There were significant improvements from baseline BCVA after treatment in both groups. No statistical differences of BCVA were observed at the same periods after treatment between two groups.

For 1 year after treatment, the total number of injections was 2.43 ± 1.04 and 2.72 ± 0.96, respectively (*P* = 0.27). In the ranibizumab group, logMAR BCVA improved from 0.63 ± 0.30 to 0.43 ± 0.27, 0.41 ± 0.37, 0.40 ± 0.39, 0.39 ± 0.43, and 0.39 ± 0.42 at 1, 2, 3, 6, and 12 months post-treatment, respectively, and statistically significant differences were observed between pretreatment and all post-treatment BCVA values (all *P* < 0.05). In the bevacizumab group, BCVA improved from 0.67 ± 0.28 to 0.52 ± 0.31, 0.49 ± 0.31, 0.47 ± 0.31, 0.42 ± 0.32, and 0.46 ± 0.43 at 1, 2, 3, 6, and 12 months post-treatment, respectively, and statistically significant differences were also observed between pretreatment and all post-treatment BCVA values (all *P* < 0.05). When BCVA values of the same period were compared between the 2 groups, no statistically significant differences were observed (*P* = 0.22, 0.36, 0.43, 0.71, and 0.52 at 1, 2, 3, 6, and 12 months post-treatment, respectively) (Figure 1).

**Figure 2:**
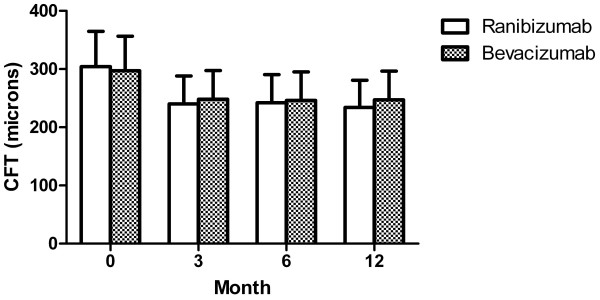
**Change of CFT after treatment of ranibizumab and bevacizumab for myopic choroidal neovascularization.** Graph shows changes of central foveal thickness (CFT) in optical coherence tomography from baseline to 12 months from treatment in both groups. There were significant decreases from baseline CFT after treatment in both groups. No statistical differences of CFT were observed at the same periods after treatment between two groups.

Seventeen eyes in the ranibizumab group and 32 eyes in the bevacizumab group that underwent OCT examination at pre-treatment and 3, 6, and 12 months post-treatment were included in the analysis of CFT. Pretreatment CFT of the ranibizumab and bevacizumab groups was 304 ± 76 and 297 ± 62 μm, respectively (*P* = 0.72). In the ranibizumab group, CFT decreased by 20.21%, 19.58%, and 22.43% at 3, 6, and 12 months post-treatment, respectively, and statistically significant differences were observed between pretreatment and all post-treatment CFT values (all *P* < 0.05). In the bevacizumab group, CFT decreased by 15.20%, 15.67%, and 15.56% at 3, 6, and 12 months post-treatment, respectively, and statistically significant differences were observed between pretreatment and all post-treatment CFT values (all *P* < 0.05). When the rates of decreased CFT at the same period were compared between the 2 groups, no statistically significant differences were observed (*P* = 0.25, 0.33, and 0.15 at 3, 6, and 12 months post-treatment, respectively). When the absolute CFT values at the same period were compared, there were also no statistically significant differences between the 2 groups (*P* = 0.65, 0.77, and 0.41 at 3, 6, and 12 months post-treatment, respectively) (Figure 2).

The proportions of patients who gained or lost 2 or more lines of Snellen BCVA and in whom CFT decreased or increased by 10% or more at 6 and 12 months after treatment are summarized in Table 2. At 6 month follow up, BCVA improved by 2 or more lines in 18 of 23 eyes (78%) and 27 of 43 eyes (63%) in each group (*P* = 0.24). Only 1 eye in each group experienced loss of 2 or more lines of BCVA despite the intravitreal injections (*P* = 0.99). At 12-month follow up, BCVA improved by 2 or more lines in 17 of 23 eyes (74%) and 27 of 43 eyes (63%) in each group (*P* = 0.43) and worsened in 1 of 23 eyes and 2 of 43 eyes in the groups (*P* = 0.99). At 6-month follow up, CFT decreased by 10% or more in 13 of 17 eyes (77%) and 24 of 32 eyes (75%) in each group (*P* = 0.91) and increased by 10% or more in only 1 eye in the bevacizumab group (*P* = 0.99). At 12-month follow up, CFT decreased by 10% or more in 14 of 17 eyes (82%) and 22 of 32 eyes (69%) in each group (*P* = 0.50) and increased by 10% or more in 2 of 32 eyes (6%) in the bevacizumab group (*P* = 0.54).

**Table 2:** Ranibizumab versus bevacizumab for myopic CNV: BCVA and CFT changes at month 6 and 12 after treatment

	**Ranibizumab**	**Bevacizumab**	***P *** value*
BCVA changes at month 6			
Gained ≥ 2 Lines	18/23 (78%)	27/43 (63%)	0.24
Lost ≤ 2 Lines	1/23 (4%)	1/43 (2%)	0.99
BCVA changes at month 12			
Gained ≥ 2 Lines	17/23 (74%)	27/43 (63%)	0.43
Lost ≤ 2 Lines	1/23 (4%)	2/43 (5%)	0.99
CFT changes at month 6			
Decreased by 10% or more	13/17 (77%)	24/32 (75%)	0.91
Equal	4/17 (23%)	7/32 (22%)	
Increased by 10% or more	0/17 (0%)	1/32 (3%)	0.99
CFT changes at month 12			
Decreased by 10% or more	14/17 (82%)	22/32 (69%)	0.50
Equal	3/17 (18%)	8/32 (25%)	
Increased by 10% or more	0/17 (0%)	2/32 (6%)	0.54

*CNV* choroidal neovascularization; *BCVA* best-corrected visual acuity; *CFT* central foveal thickness.

*Pearson’s chi-square test was used.

FA revealed leakage at the late phase in 4 of 22 eyes in the ranibizumab group and 8 of 31 eyes in the bevacizumab group at 3-month follow up (*P* = 0.74), 1 of 22 eyes and 3 of 34 eyes at 6-month follow up (*P* = 1.00), and 1 of 19 eyes and 4 of 33 eyes at 12-month follow up (*P* = 0.64). Myopic CNV recurred in 1 of 23 eyes in the ranibizumab group and 5 of 43 eyes in the bevacizumab group during 12-month follow up (*P* = 0.66, Fisher’s exact test). The former recurred at 9 months after treatment, and an additional intravitreal ranibizumab injection was performed. The latter all recurred at 12 months after treatment, and additional bevacizumab injections were performed. No severe complications such as endophthalmitis or increased intraocular pressure occurred in either group over the follow-up period.

## Discussion

The introduction of anti-VEGF agents such as ranibizumab and bevacizumab has made ophthalmologists consider it the first-choice treatment for various retinal disorders. Several multi-center, randomized, prospective studies have demonstrated anti-VEGF agents to be an effective treatment option for diabetic macular edema, retinal vein obstruction macular edema, and exudative age-related macular degeneration (AMD) [19-22]. In myopic CNV, although no large-scale, multi-center, randomized study has demonstrated the efficacy of anti-VEGF agents to date, many small scale studies have indicated that intravitreal anti-VEGF agent injection can improve functional and anatomical visual outcomes [5-13]. Recently, Ruiz-Moreno et al. reported that the improvement of visual outcomes maintained in 4 year follow-up in myopic CNV patients treated with anti-VEGF agents [17].

Both ranibizumab and bevacizumab are humanized murine monoclonal antibodies against VEGF, but there are some differences between them. The former consists of a 49-kD Fab fragment of antibody; the latter is a 149-kD whole antibody. Ranibizumab may have faster retinal penetration in comparison with bevacizumab due to its smaller molecular size, which would aid in approaching the lesion [23,24]. Ranibizumab is also known to have a higher affinity to VEGF [25]. However, the larger size of bevacizumab may guarantee a longer duration of action. In a multicenter, single-blind, prospective study to compare ranibizumab and bevacizumab in 1,208 patients with exudative AMD, the two agents were revealed to have similar treatment effectiveness when intravitreally injected according to the same protocols [26].

In our study, we demonstrated that the two agents led to similar functional and anatomical visual improvement in the 12-month follow-up period when injected as needed after the first intravitreal injection. LogMAR BCVA improved markedly at 1 month after the first injection in both groups, and it was maintained similarly over the follow-up period (Figure 1). The degree of improvement in BCVA at 12 months after intravitreal injections was similar to those reported in previous studies [6-13]. CFT also improved similarly in both groups. CFT decreased significantly at 3 months after injection in both groups, and it maintained this improvement over the follow-up period. Although we could not estimate CFT for all patients at 1 and 2 months post-treatment due to the retrospective nature of this study, the patterns of BCVA improvement strongly suggest that these values would also decrease markedly from baseline CFT. The amount of CFT decrease at 12 months after intravitreal injection was similar to those reported in previous studies, although the absolute values of CFT in the pre- and post-treatment periods were higher than in these studies [6,9,11,12]. The difference is thought to arise from differences in OCT instruments. We used the Spectralis or Cirrus OCT in 39 of 66 eyes, and these modalities are known to estimate retinal thickness more thickly than Stratus OCT [27].

To date, four clinical studies have compared treatment effectiveness after intravitreal injection of ranibizumab and bevacizumab in myopic CNV [14-17]. Gharbiya et al. [14] found that ranibizumab and bevacizumab had similar treatment efficacy in myopic CNV, although the study was limited due to short follow-up. Ruiz-Moreno et al. documented similar visual improvements with the two agents over a 4-year follow-up period in a retrospective study including 92 myopic CNV patients, although the study did not analyze anatomical changes using OCT [17]. Iacono et al. reported that the two intravitreal drugs are effective similarly in the treatment of subfoveal myopic CNV over an 18-month follow-up [16]. In this randomized, prospective study, BCVA (logMAR) improved from 0.59 ± 0.32 to 0.40 ± 0.38, from 0.61 ± 0.28 to 0.44 ± 0.32 at 18 months after treatment in each group, which were similar to our results. However, the mean number of injections was 4.72 ± 2.24 in bevacizumab group and 2.56 ± 1.61 in ranibizumab group, and they insisted that ranibizumab seemed to achieve a slightly greater efficacy than bevacizumab [16]. There were no statistical differences in the number of injections between two drugs in other studies including ours, although 3 studies were retrospective studies [15,17]. The additional intravitreal injection is usually needed to treat a recurring CNV lesion as well as to diminish a naïve lesion, although Iacono et al. [16] did not explain why the patients in bevacizumab group needed more intravitreal injections. The Comparison of Age-related Macular Degeneration Treatment Trial (CATT) study [26] also revealed that intravitreal ranibizumab and bevacizumab injection had a similar effect in exudative AMD when the same protocol was applied and the mean number of injections was similar between two groups (6.9 and 7.7, as needed protocol). Compared with exudative AMD, myopic CNV usually involves a smaller lesion with minimal retinal edema and often accompanies lacquer crack, a rupture of the Bruch membrane. Although no experimental study comparing their pharmacokinetics in high myopia has been reported, these environments would seem to make it easier for anti-VEGF agents to have access to a lesion, ignoring the pharmacologic difference between ranibizumab and bevacizumab. Our results indicated that bevacizumab and ranibizumab have similar effects in the treatment of myopic CNV.

Our study has a few drawbacks. As a retrospective study, it has intrinsic limitations regarding bias control. The patients selected which drugs are injected under sufficient explanation, which would be a selection bias. However, any statistically significant differences were not found in demographic data between two groups. We calculated retinal thickness using several OCT modalities, making comparisons of retinal thickness between patients are less meaningful. However, only one OCT modality was applied to each patient during the follow-up period, which made comparisons of CFT between pre- and post-treatment period reasonable.

## Conclusions

In conclusion, we showed that in a retrospective study, ranibizumab and bevacizumab had similar functional and anatomical effectiveness for myopic CNV over a 12-month follow-up period. Given that this conflicts with previous results from Iacono et al. [16], some additional questions remain. A further prospective study controlling explicitly for number of injections across the two groups could yield more conclusive findings.

## Abbreviations

CNV: Choroidal neovascularization; PDT: Photodynamic therapy; VIP: Verteporfin in photodynamic; anti-VEGF: Anti-vascular endothelial growth factor; BCVA: Best-corrected visual acuity; FA: Fluorescein angiography; OCT: Optical coherence tomography; CFT: Central foveal thickness; logMAR: Logarithm of the minimum angle of resolution; AMD: Age-related macular degeneration; CATT: Comparison of age-related macular degeneration treatment trial.

## Competing interests

The authors declare that they have no competing of interests.

## Authors’ contributions

CDM the statistical analyses, acquisition of data and preparation of the first draft of the manuscript. KTW study concept and design, interpretation of data, drafting/revising the manuscript. WSJ, HJW, PKH, YHG, and CH Review and approval of the manuscript. All authors read and approved the final manuscript.

## Pre-publication history

The pre-publication history for this paper can be accessed here:

http://www.biomedcentral.com/1471-2415/14/69/prepub
